# Are QM/MM models systematically improvable? A case study on carbonic anhydrase binding affinities

**DOI:** 10.1007/s10822-026-00954-8

**Published:** 2026-10-07

**Authors:** Haedam Mun, Junming Ho

**Affiliations:** School of Chemistry, The University of New South Wales, Sydney, NSW 2052 Australia

**Keywords:** QM/MM, Carbonic anhydrase, Binding free energy, Cluster models, Molecular docking, Solvent effects

## Abstract

Hybrid quantum mechanics/molecular mechanics (QM/MM) is the method of choice for modelling large biochemical systems, but their performance depends critically on how they are implemented. This paper examines the sensitivity of QM/MM enzyme-ligand binding energy predictions to key parameters, including QM region size, the incorporation of thermal and solvation effects as well the use of docked poses versus crystal structures. The focus is on carbonic anhydrase which has emerged as an important drug target for a wide variety of pathologies. It is demonstrated that trends in binding affinities are surprisingly insensitive with respect to these parameters. QM/MM geometry optimization of poses obtained from molecular docking enhances predictive utility for yet-to-be synthesized ligands, though outliers arise from structural deviations or non-native interactions. These findings provide practical guidelines for QM/MM applications in carbonic anhydrase and similar metalloenzymes.

**Graphical Abstract:**

**Figure:**
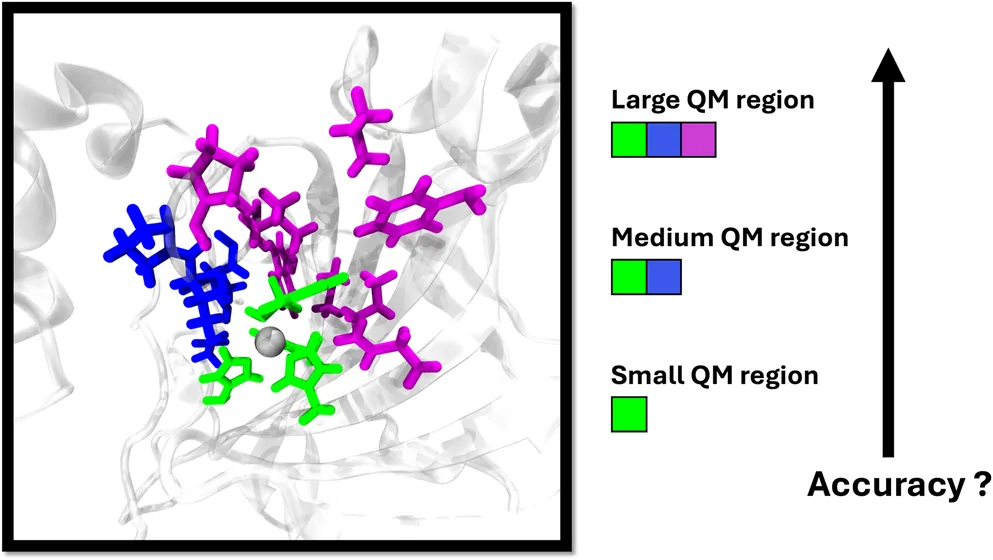

**Supplementary Information:**

The online version contains supplementary material available at https://doi.org/10.1007/s10822-026-00954-8.

## Introduction

Hybrid quantum mechanics/molecular mechanics (QM/MM) models are the method of choice for computational studies of enzyme and condensed-phase reactions. For large systems where direct QM treatments are impractical, these methods partition the system into two or more subsystems that are treated with methods of different accuracy and cost. Typically, the region of the system deemed most important for the property of interest (e.g. the enzyme active site) is treated using rigorous quantum chemical methods, and the remaining atoms and their interactions with the QM subsystem are described using approximate (usually classical) methods.

This appears to be a very successful strategy, where QM/MM methods have been widely used to study molecular properties and the mechanisms of (bio)chemical reactions in enzymes [1–5], solvents [6–8], and interfaces [9, 10]. However, the implementation of QM/MM models is far from trivial. This is because there are numerous parameters such as the pairing of QM and MM potentials, the QM region size and composition, the treatment of conformational sampling and QM-MM boundaries. Furthermore, the optimal combination of these parameters will likely differ for different systems [11], so some level of benchmarking is usually needed depending on the level of accuracy sought by the user. Indeed, even experts cannot agree on how best to implement these models and seemingly minor variations in the simulation protocol can lead to large differences in the calculated reaction thermochemistry [12–14]. 

QM/MM modelling may be further divided into static versus dynamics approaches [15]. This paper is concerned with the former approach where QM/MM calculations are performed on a crystal structure, poses obtained from molecular docking or a relatively small number of configurations sampled from molecular dynamics (MD) simulations. The advantage of this “single-structure approach” is that one can afford to use very accurate quantum chemistry methods to treat potentially very large QM regions and thermal and solvation effects can be separately recovered using the rigid rotor (quasi)harmonic oscillator approximation.

A key question that we seek to answer in this paper is whether QM/MM model predictions can be systematically improved. Specifically, we are interested in predicting human carbonic anhydrase (hCA)-ligand binding free energies, as this Zn(II) metalloenzyme has emerged as an important drug target for the treatment of serious diseases, including certain types of cancer [16–18]. The findings from this work may be transferable to modelling of other related enzymes such as matrix metalloproteinases (MMPs [19]) and histone deacetylase (HDACs [20]), which are also important therapeutic targets. Existing computational approaches, such as molecular docking, classical MD simulations, and QM-cluster models, have limited predictive power in large-scale tests [21, 22]. For example, our group and others have employed classical molecular dynamics simulations in conjunction with methods such as MM/GBSA [21, 23] or umbrella sampling [24] to predict ligand binding free energies with hCA isoform II. Moderately strong correlations with experimental binding energies have been reported (*R*^2^ = 0.40–0.80) depending on the test set of ligands. Molecular docking using contemporary force fields optimized for Zn(II) such as Autodock Vina [25, 26] and Autodock4_Zn_ force field [27] yielded good correlations for small datasets of structurally similar sulfonamide ligands, but their performance deteriorates when diverse ligands are considered [23]. 

**Fig. 1:**
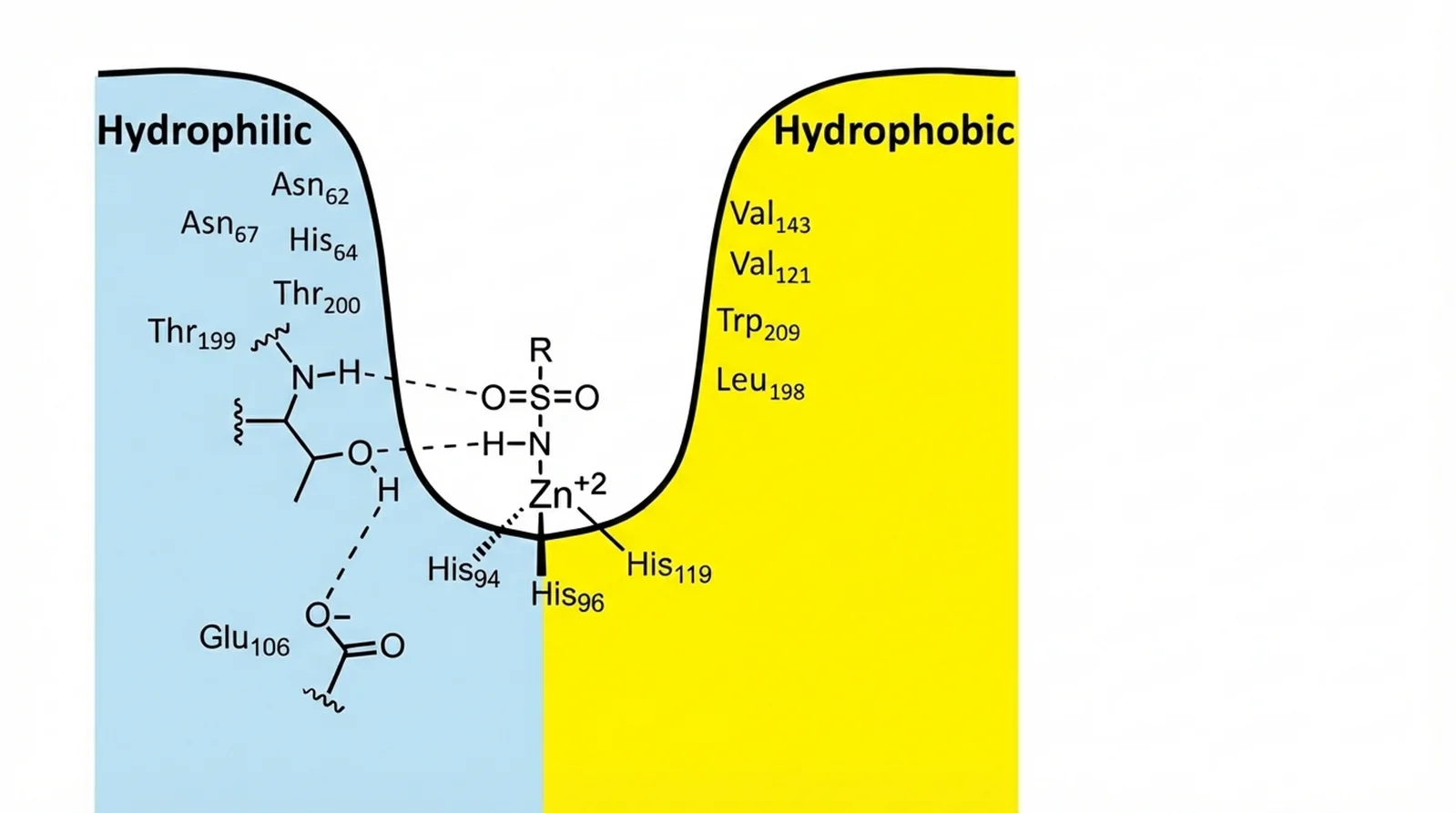
Active site of hCA II characterized by a conical cleft split into hydrophobic and hydrophilic sides. Thr_199_ is known to form hydrogen bonds with the sulfonamide head group

Thus, it is of interest to examine if QM/MM models can offer an improvement here. Notably, hCA is a zinc(II) metalloenzyme and it is challenging for classical force fields to describe metal-ligand and metal-protein interactions due to effects such as charge transfer, polarization and variable coordination geometries [28, 29]. Our recent work on QM-cluster models demonstrated that it is possible to achieve moderate correlation (*R*^2^ of up to 0.67 cf. 0.37 for docking) in predicted CA binding affinities when considering a test set of small to medium-sized ligands [22]. Fig. 1 depicts the binding pocket of hCA which is characterized by a 15 Å deep conical cleft that is split into the hydrophilic and hydrophobic walls, with important residues such as Thr199 that are crucial for binding sulfonamide inhibitors.

In our QM-cluster models, the binding pocket was represented by the active site and a small number of proximal residues (up to 16 in the largest model). While these models permit the use of accurate quantum chemistry methods (DLPNO-CCSD(T)/CBS quality) to interrogate the interactions at the active site, the broader enzyme environment as well as thermal and solvent effects were neglected. Recovery of these effects could potentially further increase the predictive power of these models.

To this end, we first generated QM/MM models of varying QM region sizes to ensure that the model has converged. We also examined the impact of the level of theory used to generate the atomic charges of the QM atoms as well as relaxation of selected MM atoms, which allows distal residues to adjust their positions to accommodate larger ligands that have motifs that extend far from the active site. These are important considerations as earlier studies have indicated that minor variations in protocol and/or small region QM/MM models can result in substantially different reaction barriers and energies by many kcal·mol^− 1^ [14]. We built on our recent work where we identified low-cost density functional theory (DFT) methods that can reproduce benchmark DLPNO-CCSD(T)/CBS Zn(II)-ligand binding energies [22]. Notably, the ωB97M-V/def2-TZVPP level of theory showed the best agreement (0.5 kcal·mol^− 1^ on average) and the r^2^SCAN-3c composite method represented a cost-effective alternative with ~ ten-fold speedup [22]. 

All QM/MM and QM-cluster models are based on a “single-structure” approach [30], i.e. without configurational sampling, to facilitate comparison between the two models. In our previous work [22], we analyzed the side-chain conformation of 16 key residues in the binding pocket across crystal structures of 23 ligands. It was observed that the rotamers are highly conserved across the diverse set of ligands and therefore we did not consider alternative rotamers in our models. The limitation of this approximation is discussed later in the paper. With a converged QM/MM model in hand, we then systematically examined how the inclusion of thermal and solvent effects impacts the agreement between calculated and experimental binding free energies. Finally, the study examined how the use of binding poses obtained from molecular docking in place of crystal structure poses impacts accuracy. This is especially important for gauging the predictive power of our models in situations where such structural data is unavailable (e.g. for yet-to-be-synthesized ligands).

**Fig. 2:**
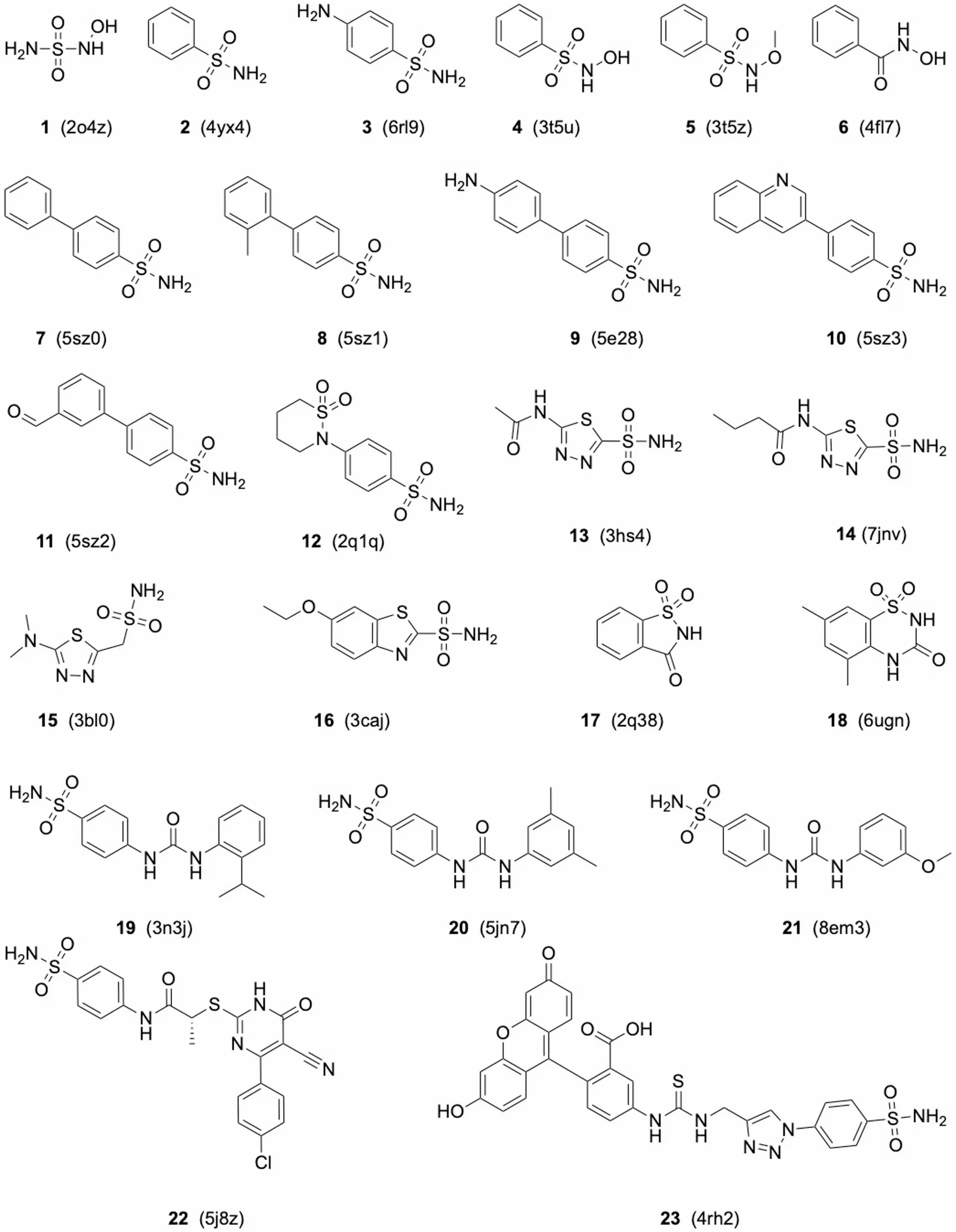
Chemical structures of ligands in the test set with their crystal structure PDB ID in parentheses

Figure 2 presents the chemical structures of the test set of 23 diverse ligands (mostly sulfonamides) examined in this work. This test set is identical to the one used in our recent QM-cluster model study [22] and allows direct comparison with the QM/MM models. Note that ligands **19** to **23** are larger and conformationally flexible sulfonamides that were deliberately added to the test limits of the QM-cluster models, and in this study, the QM/MM models.

This dataset has been carefully curated based on the availability of high-resolution crystal structures (average resolution of 1.6 Å) of their enzyme-bound complexes as well as measurements of the binding constants using a consistent technique (stopped-flow CO_2_ hydration assay) [31]. This is critical as different techniques have been shown to yield very different binding affinities (opposite trends) in some cases [32]. We assessed the accuracy of our QM/MM models in terms of their ability to reproduce the trends in experimental binding free energies using the coefficient of determination (hereafter referred to as *R*^2^). From an experimental point of view, this is a pragmatic metric for evaluating accuracy as relative binding energies are especially useful for prioritizing synthesis.

### Computational details

All electronic structure calculations were carried out using the Gaussian 16 [33], ORCA 5.0.4 [34], and xTB [35] programs. The gas-phase lowest energy conformers of the free ligands were obtained using the CREST [36] and CENSO [37, 38] programs where conformers were sampled from metadynamics simulations, optimized with GFN2-xTB, followed by sequential ranking with B97-D/def2-SVP and r^2^SCAN-3c single point energies followed by geometry optimization at the r^2^SCAN-3c level.

A high resolution (0.9 Å) crystal structure of a sulfonamide-bound hCA II enzyme (PDB ID: 3K34) [39] was used for the preparation of the initial enzyme-ligand complex. The H++ server [40–42] was used to add missing hydrogens, assuming a pH of 6.5 and internal and external dielectric constants of 10 and 80 (default values), respectively. The ambpdb tool from AmberTools22 [43] was used to generate the final protonated enzyme structure. Restrained electrostatic potential (RESP) [44] atomic charges for the ligand were generated with the general Amber force field (GAFF) using Antechamber [45]. The Zn(His)_3_ charges were fitted using espgen and resp tools in AmberTools22. Different levels of theory (HF and B3LYP in conjunction with 6-31G(d), 6–31+G(d), and 6–31+G(d,p) basis sets) were used to calculate the ligand RESP charges to examine their impact on the accuracy of the model predictions (*vide infra*).

The QM/MM calculations were performed using the two-layer ONIOM [46–49] method implemented in Gaussian16 and the Toolkit for Assisting ONIOM (TAO) package [50] was used to generate the ONIOM input files. The QM region was treated using the r^2^SCAN-3c [51] method for geometry and frequency calculations followed by single point calculations at ωB97M-V/def2-TZVPP level as informed by our previous benchmarking study [22]. The enzyme MM atoms were simulated using the Amber force field (parm99) in Gaussian16. As the r^2^SCAN-3c method is not currently implemented in Gaussian16, an in-house script was used to read in r^2^SCAN-3c energies and forces from ORCA (employing Tight SCF and def2grid integration grid) so that all ONIOM geometries and harmonic frequencies are computed using Gaussian 16’s optimizer.

The QM/MM geometry was prepared in several stages. First, all hydrogen atoms were initially optimized with the Amber force field with heavy atoms fixed at their crystallographic coordinates. This was followed by QM/MM optimization of the QM hydrogens with the heavy atoms held fixed. Finally, the QM/MM optimization was run with all QM atoms relaxed as well as proximal MM residues (*vide infra*) with their backbone frozen. Frequency calculations were conducted numerically using analytically calculated first derivatives from the Gaussian-ORCA interface at the same level of theory (verytightSCF and Defgrid3) using a step size of 0.001 Å. Grimme’s quasi-harmonic entropy [52] and Head-Gordon’s quasi-harmonic enthalpy correction [53] were applied through the Goodvibes [54] program with a cutoff frequency of 100 cm^− 1^.

Molecular docking was performed with the zinc optimized AutoDock4_Zn_ force field [27] in the AutoDock Vina program [26] using the enzyme geometry from the crystal structure PDB ID: 3K34. Docking was performed in triplicates for each ligand with an exhaustiveness score of 32.

**Scheme 1:**
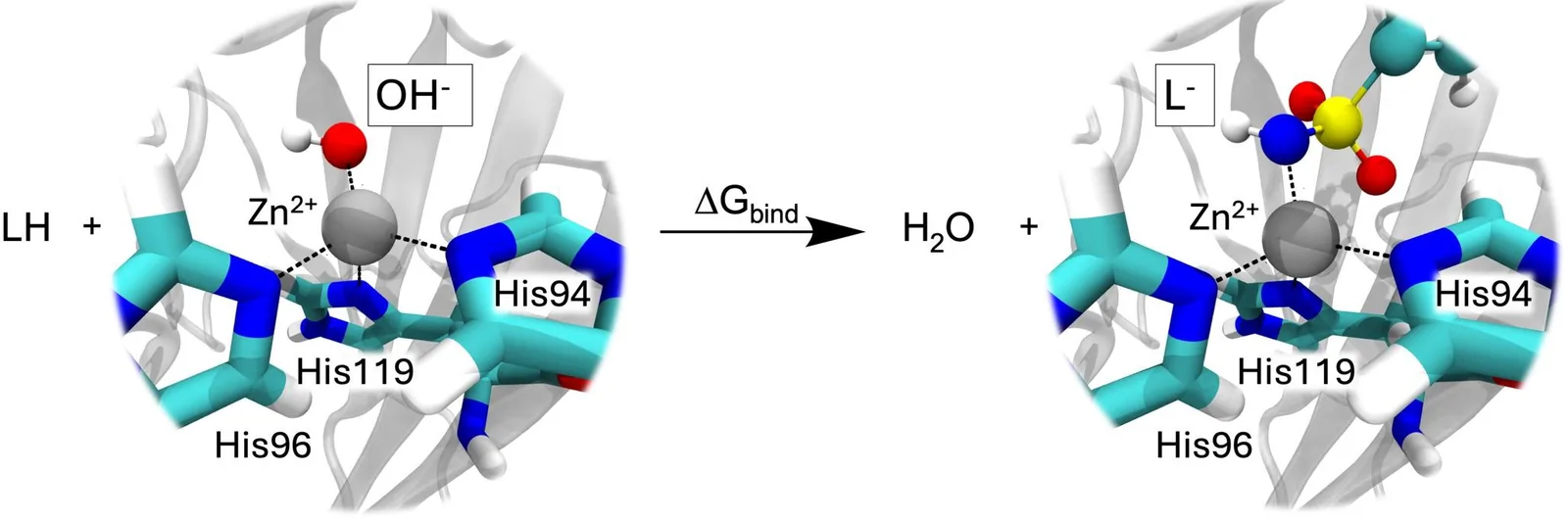
The hydroxide displacement reaction used to compute ligand binding affinities

The binding energy was modelled as the hydroxide displacement reaction shown in Scheme 1, where the ligand in its neutral form displaces the CA-bound hydroxide, forming the ligand-CA complex with the ligand bound in its deprotonated form and a water molecule [55, 56]. 

Solvation free energies were computed using the three-dimensional reference interaction site model (3D-RISM) [57, 58] implemented in the RISMiCal [59, 60] program. The Kovalenko-Hirata [61] closure equations, ff19SB [62] and GAFF2 [63] forcefields and B3LYP/6–31+G(d,p) RESP charges were used for the calculations and pressure correction built into RISMiCal was used to obtain the final solvation free energies. These are fixed concentration solvation free energies ($\:{\varDelta\:{G}^{*}}_{solv}$) denoted with an asterisk superscript “*” where the standard state of 1 M is used for both gas and aqueous phases.

The Gibbs free energy in solution is given by:

1 $$\:{{G}^{*}}_{soln}={E}_{gas}+{{G}^{\mathrm{o}}}_{corr}+{\varDelta\:{G}^{*}}_{solv}+{\varDelta\:G}^{\mathrm{o}\to\:*}$$

where $\:{{G}^{\mathrm{o}}}_{corr}$ is the thermal correction at the standard state of 1 atm, $\:{\varDelta\:{G}^{*}}_{solv}$ is the solvation free energy, and $\:{\varDelta\:G}^{\mathrm{o}\to\:*}$ represents the standard state correction.

## Results and discussion

### QM/MM model setup

We first examined several critical elements in our QM/MM protocol: (a) level of theory used to generate atomic charges of ligand and Zn(His)_3_ core, and (b) relaxation of MM atoms.

Typically, RESP charges with GAFF are generated at the HF/6-31G(d) level on corresponding optimized geometry. However, our previous work demonstrated that correlation between MM/GBSA and experimental CA binding energies are quite sensitive to the level of theory used to generate the ligand charges [21]. There is also significant charge transfer between the Zn(II) ion and coordinating histidine residues which are not adequately described by the Amber force field. Thus, the RESP atomic charges of the ligands and the Zn(His)_3_ core were generated separately at several different levels of theory, namely HF and B3LYP in conjunction with the 6-31G(d), 6–31+G(d), and 6–31+G(d,p) basis sets, to compute the zero-Kelvin binding energies (excluding zero-point vibrational energy) according to Scheme 1. The atomic charges of the rest of the enzyme are based on the Amber force field (parm99) in Gaussian 16.

The atomic charge calculations for the ligands were performed on their r^2^SCAN-3c optimized geometries. For the Zn(His)_3_ motif, its geometry was extracted from an ONIOM(r^2^SCAN-3c: AMBER) optimized geometry of the apoenzyme with the Zn(His)_3_ in the QM region (the QM/MM boundary was at the C_α_-C_β_ bonds of the histidines). The histidine residues were truncated at the C_α_-C_β_ bond and capped with hydrogens. The charges of the capping hydrogens were set to zero in the RESP charge fitting. The resulting atomic charges for two representative ligands and the Zn(His)_3_ motif are presented in Tables S1 and S2 in the Supporting Information. Overall, the atomic charges are relatively insensitive to the level of theory employed, though it was observed that the positive atomic charges on the sulfonamide sulfur and the Zn(II) ion are noticeably smaller by 0.1 to 0.2 *e* for B3LYP compared with HF. In all cases, the atomic charge of the Zn(II) ion is close to 1.0 *e*, which is consistent with the value used in previous MD studies [23, 64]. 

ONIOM(ωB97M-V/def2-TZVPP: AMBER*) single point energies were computed on the ligand and hydroxide bound enzyme where AMBER* signifies that the atomic charges of the ligand and the Zn(His)_3_ motif have been replaced with RESP charges obtained at the above-mentioned levels of theory. In these ONIOM calculations, the QM region is composed of the ligand, Zn(His)_3_ core, Thr199, Thr200, Pro201, and Glu106. The geometries were optimized at the ONIOM(r^2^SCAN-3c: AMBER*) level of theory where all QM and link atoms and side chains of MM residues within 6 Å of ligand **2** in its crystal binding pose were relaxed.

The residues in the QM region are core residues that participate in the zinc-ligand binding and form a crucial hydrogen bonding network with the sulfonamide headgroup [65]. The resulting zero-Kelvin binding energies (ΔE_bind_) are summarized in Table S4 where it is observed that the standard deviations in the binding energies computed using different sets of atomic charges are generally less than 1 kcal·mol^− 1^. More importantly, there is minimal impact on the correlation between computed and experimental binding free energies where the *R*^2^ is consistently around 0.65. In the subsequent ONIOM calculations, the ligand and Zn(His)_3_ charges generated with B3LYP/6–31+G(d,p) is used as it showed the highest correlation with experimental binding free energies (Table S4).

Before we proceed to examining the effect of QM region size, we explore the effect of relaxing selected atoms in the MM region during the QM/MM geometry optimization step. As shown in Fig. 2, the sulfonamide ligands vary significantly in size, with some ligands (e.g., **22** and **23**) that have “tails” that extend far away from the Zn(II) binding site. Appending tail groups to sulfonamide is a popular strategy used to develop isoform-selective CA binders as the active site is highly conserved across the various isoforms and distal interactions are exploited to achieve selectivity [66]. Relaxing the MM atoms in the vicinity of the binding pocket could resolve any potential steric interactions and/or allow the enzyme environment to respond to interactions with the ligand, albeit at the MM level.

**Fig. 3:**
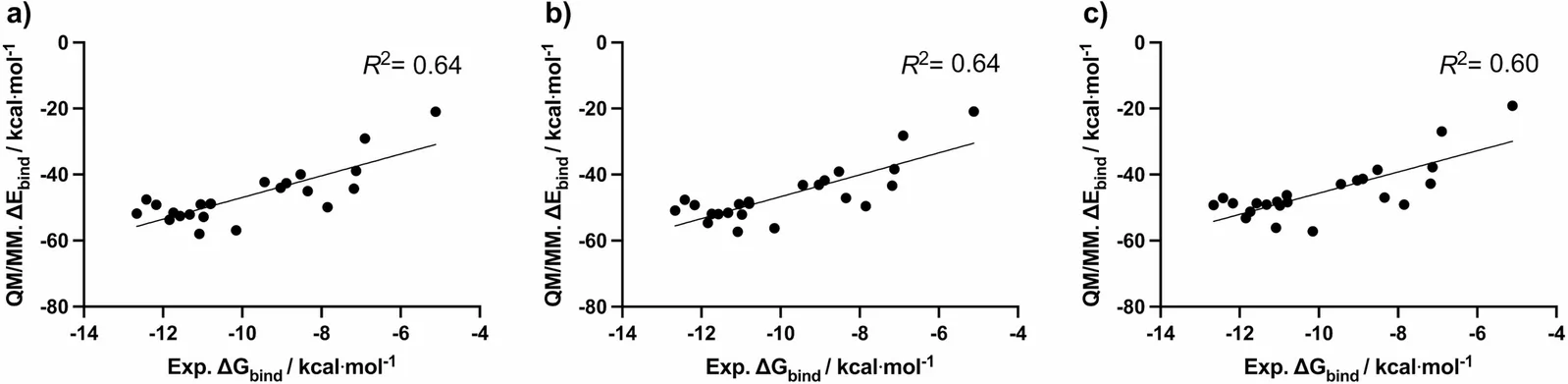
The electronic binding energies computed with (**a**) 3.5 Å around ligand **2** (**b**) 6 Å around ligand **2** (**c**) 6 Å around all ligands in the test set relaxed plotted against experimental binding affinities

**Fig. 4:**
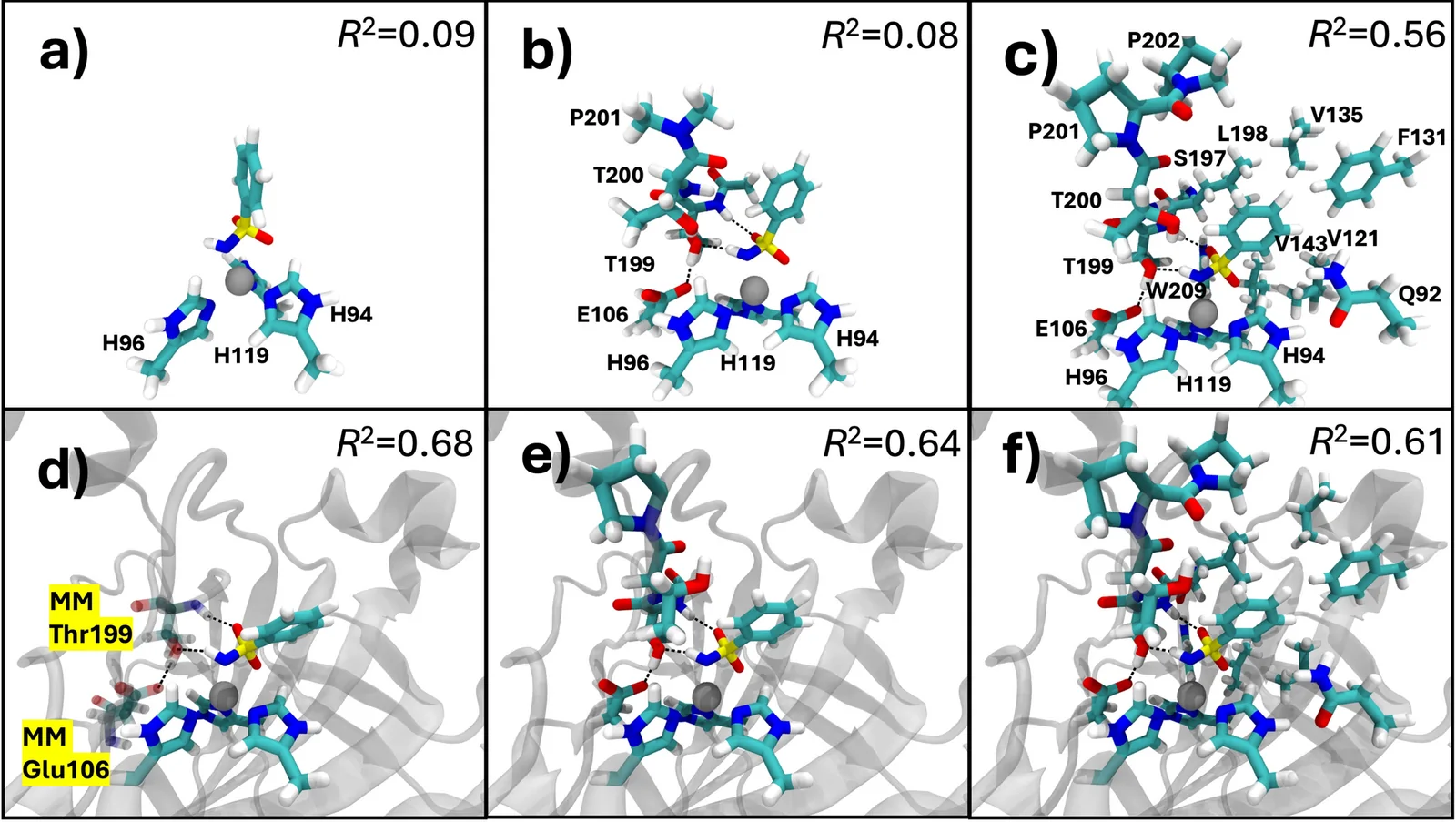
**a**-**c **The three QM-cluster models considered in previous work [22]. Panels (**d**-**f**) illustrate corresponding QM/MM models I, II, and III investigated in this study. *R*^2^ relative to experimental binding affinities are presented for each model

We considered three different criteria for selecting unconstrained MM atoms: (a) residues within 3.5 Å of ligand **2**, (b) within 6 Å of ligand **2**, and (c) within 6 Å around all ligands (based on crystal structure coordinates) in the test set. The same QM region (see Fig. 4(e)) was used and the number of relaxed residues for each selection criteria was 11, 24, and 45 and their side chains were relaxed during the geometry optimization step. The difference in the correlation between ΔE_bind_ and experimental binding energies was minimal (*R*^2^ of 0.64, 0.64 and 0.60 for criteria (a), (b), and (c), respectively) as shown in Fig. 3.

It appears that the largest relaxed region (c) displays the weakest, albeit slightly, correlation presumably because relaxing many MM residues increases the variations in the MM atom positions between ligand and hydroxide bound enzyme optimized structures (Scheme 1) which can contribute to “noise” in the calculated binding energies. To investigate this possibility, we decomposed the ONIOM reaction energy according to the QM and MM contributions:

2 $$\:\Delta\mathrm{E}\left(\mathrm{QM}\right)\:=\:\mathrm{E}^\mathrm{QM}(\mathrm{Enz}-\mathrm{L})\:-\:\mathrm{E}^\mathrm{QM}(\mathrm{E}\mathrm{n}\mathrm{z}-\mathrm{O}\mathrm{H})$$

3 $$\:\Delta\mathrm{E}\left(\mathrm{M}\mathrm{M}\right)\:=\:\mathrm{E}^\mathrm{MM}(\mathrm{E}\mathrm{n}\mathrm{z}-\mathrm{L})\:-\:\mathrm{E}^\mathrm{MM}(\mathrm{E}\mathrm{n}\mathrm{z}-\mathrm{O}\mathrm{H})$$

3a $$\:\mathrm{E}^\mathrm{MM}(\mathrm{E}\mathrm{n}\mathrm{z}-\mathrm{L})\:=\:\mathrm{E}^\mathrm{MM}\left(\mathrm{r}\mathrm{e}\mathrm{a}\mathrm{l}\right)\:-\:\mathrm{E}^\mathrm{MM}\left(\mathrm{m}\mathrm{o}\mathrm{d}\mathrm{e}\mathrm{l}\right)$$

3b $$\:\mathrm{E}^\mathrm{MM}(\mathrm{E}\mathrm{n}\mathrm{z}-\mathrm{O}\mathrm{H})\:=\:\mathrm{E}^\mathrm{MM}\left(\mathrm{r}\mathrm{e}\mathrm{a}\mathrm{l}\right)\:-\:\mathrm{E}^\mathrm{MM}\left(\mathrm{m}\mathrm{o}\mathrm{d}\mathrm{e}\mathrm{l}\right)$$

4 $$\Delta\mathrm{E}(\mathrm{Q}\mathrm{M}/\mathrm{M}\mathrm{M})=\Delta\mathrm{E}\left(\mathrm{Q}\mathrm{M}\right)+\Delta\mathrm{E}\left(\mathrm{M}\mathrm{M}\right)\:+\mathrm{E}^\mathrm{QM}\left(\mathrm{H}_{2}\mathrm{O}\right)\:-\:\mathrm{E}^\mathrm{QM}\left(\mathrm{L}\mathrm{H}\right)$$

Here, Enz-L and Enz-OH refer to the enzyme complex with the ligand and the hydroxide anion; real and model refer to the full system and QM subsystem respectively, and LH is the ligand. Table S3 presents the ΔE(MM) values for the 23 ligands as a function of the relaxed MM region size. Interesting, relaxing more MM atoms around the binding pocket actually decreased the standard deviation in ΔE(MM) values across the 23 ligands. This suggests that the expanded relaxed MM region indirectly increased the QM degrees of freedom, i.e. the increased noise is due to ΔE(QM), and might explain the slightly weaker correlation between computed and experimental binding energies (Fig. 3c).

Accordingly, all subsequent QM/MM calculations are based on optimized geometries where MM residues within 3.5 Å of ligand **2** are relaxed, and using ligand and Zn(His)_3_ RESP charges obtained at the B3LYP/6–31+G(d,p) level of theory.

### Effect of QM region size

QM region size and composition are important considerations when implementing QM/MM models. Previous studies have shown that calculated properties such as reaction barriers and energies can vary significantly depending on how the QM subsystem is defined [13, 67, 68]. As a side note, QM region sensitivity is a symptom of the compatibility between the MM force field and QM method used. When the MM force field is of comparable quality to the QM method, then the performance of the QM/MM model becomes independent of how the QM region is defined [15]. Fig. 4(a) to (c) depict three of the QM-cluster models considered in our earlier work [22]. These cluster models vary in size and were generated by including an increasing number of residues based on the frequency of their presence within 4.5 Å of ligands **1**–**18** in their crystal structure binding pose. We generated corresponding QM/MM models of these cluster models shown in Fig. 4(d) to (f) (referred to as QM/MM model I, II, and III, respectively) to examine whether consideration of the enzyme environment improves predictive performance. The same QM levels of theory are used in both models (ωB97M-V/def2-TZVPP for energies and r^2^SCAN-3c for geometry).

It was observed that the correlation between calculated ΔE_bind_ with experimental binding free energies improves systematically as the size of the QM-cluster model increases. The smallest QM-cluster model (Fig. 4(a)) produced an *R*^2^ of 0.09 and an incorrect slope (inverse relationship between ΔE_bind_ and experimental ΔG_bind_). The correct positive slope was restored, although weakly (*R*^2^ of 0.08), when residues Thr199, Thr200, and Glu106 were included in cluster model (b) which are part of the highly conserved hydrogen bonding network known to be important for anchoring sulfonamide inhibitors at the active site (see Fig. 1). The largest QM-cluster model (c) composed of 16 residues yielded a further improvement with *R*^2^ of 0.56 [22]. 

Expectedly, the QM/MM models significantly improved correlations compared to their corresponding QM-cluster models. The most significant improvement was observed between QM/MM model I and QM-cluster model (a), with *R*^2^ of 0.68 and 0.09, respectively. The largest improvement displayed by QM/MM model I relative to its cluster model appears to be due to its ability to reproduce the highly conserved hydrogen bonding network involving Thr199, Thr200 and Glu206. However, it is also interesting to note that the larger QM/MM models II and III did not lead to further improvement compared with the smallest QM/MM model I. Instead, there is a slight decrease in *R*^2^ with larger QM region size (Fig. 4).

**Fig. 5:**
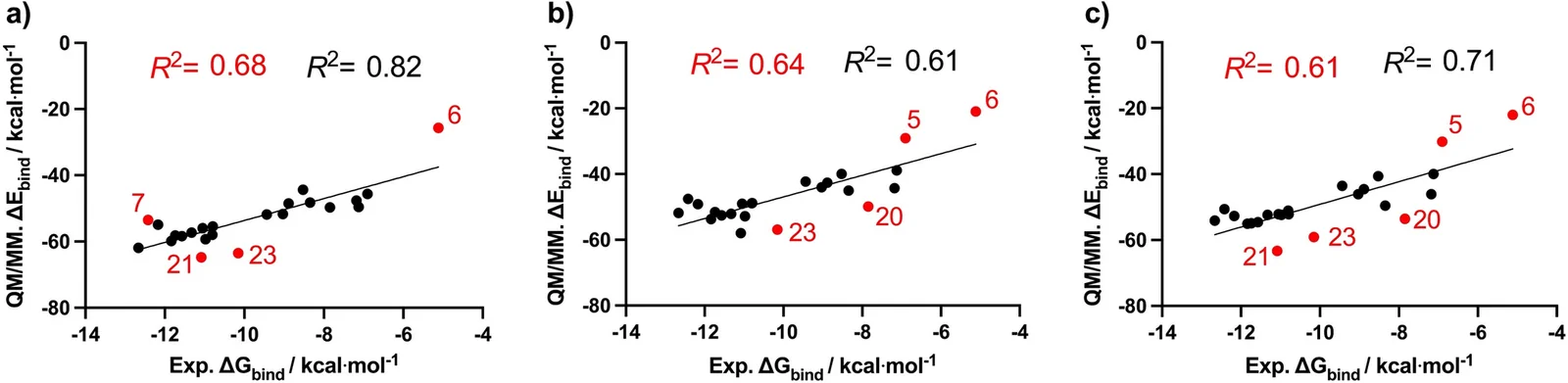
Scatter plots of QM/MM binding energies against experimental binding free energies for the three QM/MM models. **a** QM/MM model I (**d**). **b** II. **c** III. The *R*^2^ value in red includes all 23 binding affinities and *R*^2^ value in black represents data points with the red outliers removed

This is surprising as it is generally expected that the accuracy of a QM/MM model would improve as more of the ligand-enzyme interactions are modelled using accurate quantum chemistry (through increasing the QM region size). Inspection of the scatterplots of QM/MM versus experimental binding energies in Fig. 5 indicates that the trend in the correlation coefficients may be skewed by several outliers. Notably, removal of outliers (defined as data points with deviations larger than two times the mean absolute deviation from the fitted trend line) resulted in *R*^2^ of 0.82, 0.61 and 0.71 for QM/MM models I, II, and III, respectively. The lack of improvement in *R*^2^ with increasing QM region size is presumably due to the same effect of expanding the relaxed MM region (see Eqs. 2, 3, 3a, 3b and 4 and Table S3) which introduces more noise due to the increased QM degrees of freedom (all QM atoms are unconstrained).

**Fig. 6:**
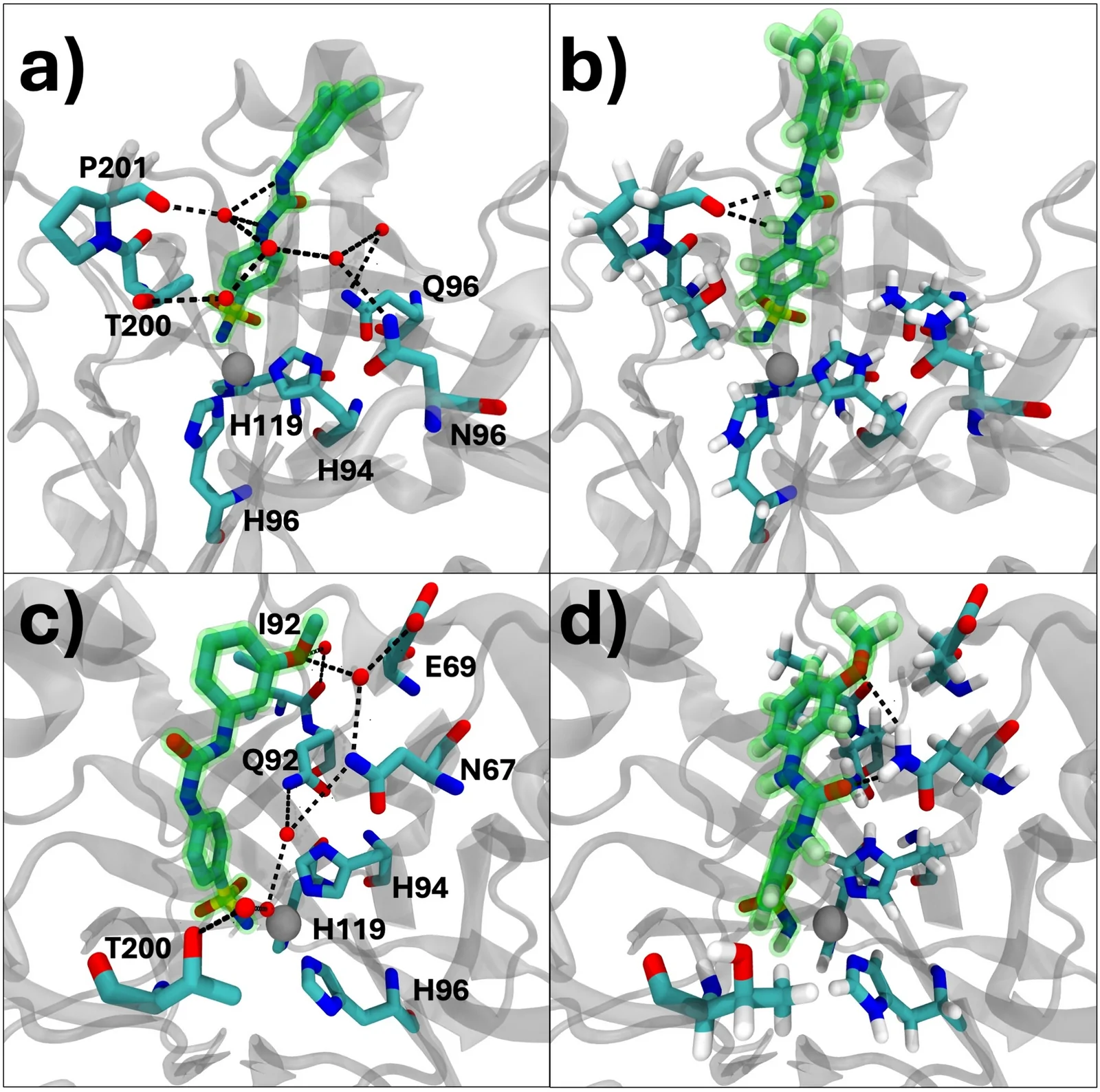
**a** Crystal structure of ligand **20** (PDB ID: 5JN7). **b** QM/MM optimized geometry of ligand **20**. **c** Crystal structure of ligand **21** (PDB ID: 8EM3). **d** QM/MM optimized geometry of ligand **21**. Crystal water molecules are represented as red spheres and the ligands are highlighted in green

The common outliers are ligands **5**, **6**, **20**, **21** and **23**. Ligand **5** is an *N*-methoxy-benzenesulfonamide while **6** is a hydroxamate (R-CONHOH), so the two ligands are chemically distinct functional groups from the rest of the test set which is dominated by sulfonamides. As noted in our previous QM-cluster model work [22], the conformationally flexible ligands **20**–**23** were also outliers. This was attributed to the single structure approach which neglects configurational entropy effects or distinct orientational states of the ligands within the binding pocket. These ligands contain a dual hydrogen bonding (thio)urea motif, and it has been observed in their crystal structures that there are water molecules bridging their interactions with the enzyme which are absent in our QM/MM models. Figure 6 compares the ONIOM(r^2^SCAN-3c: AMBER*) optimized geometries (QM/MM model III) of ligands **20** and **21** with their crystal structures.

The absence of the crystal waters resulted in the QM/MM optimized binding poses that differ significantly from the crystal structure (the RMSD of the relaxed atoms is 0.6 and 0.8 Å for ligands **20** and **21**, compared with the average of 0.3 Å for the remaining ligands). Presumably, this explains why these ligands are outliers. Inclusion of crystallographic water molecules could potentially remedy this problem, but this complicates the binding affinity calculation using Scheme 1 as the number and placement of water molecules differ for each ligand including the native hydroxide ligand.

Overall, it appears that increasing the QM region size in QM/MM models did not necessarily improve the correlation with experiment (even after removal of outliers). Our analysis uncovered greater fluctuations in the enzyme QM atom positions (relative to the averaged structure over 23 ligands and OH) nearly three times larger in QM regions (e) and (f) compared to (d). The increased degrees of freedom associated with larger relaxed QM regions may have introduced more noise in the computed binding energies, leading to the lower correlation with experiment.

We have also examined the effect electrostatic embedding (EE) compared with mechanical embedding (ME). While EE is generally considered to be physically more rigorous as it allows the QM atoms to be polarized by the MM charges, we observed a slight decrease in *R*^2^ compared to mechanical embedding (ME) (see Table S5). For example, the *R*^2^ values are 0.70 and 0.64 for QM/MM model I with ME and EE respectively. The poorer performance of EE may be due overpolarization effects when the QM/MM boundary is close to the enzyme active site, even with scaled-charge schemes [8]. This is supported by our findings that the discrepancy in *R*^2^ between ME and EE is most pronounced in the smallest QM/MM model I and diminishes as the QM region expands. Furthermore, analysis of the Mulliken charges revealed that the Zn(II) charges from the ME and EE calculations differ by 0.05 *e* on average for QM/MM model I, while the difference is only 0.02 *e* for models II and III. As such, all calculations presented in this manuscript are based on the ONIOM-ME approach. In the next section we examined how the inclusion of the thermal and solvent effects affects the QM/MM model performance.

### Consideration of thermal effects

The QM and QM/MM binding energies presented in the previous sections refer to zero-Kelvin values (Δ*E*_bind_) not including zero-point vibrational correction nor thermal effects arising from changes in vibrational, rotational and translational motion. Since the experimental binding affinities of the ligands were measured at room temperature [69–71], we have employed the rigid rotor quasi-harmonic oscillator (RR-QHO) approximation to determine the thermal correction to the Gibbs free energy (*G*_corr_ in Eq. 1) for each species appearing in Scheme 1. For the ligand and hydroxide bound enzyme, the thermal correction was computed for the QM atoms at the ONIOM(r^2^SCAN-3c: AMBER*) level of theory employing QM/MM model II. A cutoff frequency of 100 cm^− 1^ was applied to mitigate the effects of low-frequency vibrational modes on the entropic contributions.

As indicated in Fig. 5, the calculated zero-Kelvin binding energies significantly over-estimate the experimental binding energies by 36 kcal·mol^− 1^ on average. The inclusion of thermal effects (Δ*E*_bind_ + Δ*G*_corr_; see Eq. 1) reduced this deviation to 30 kcal·mol^− 1^, primarily due to the loss of translational and rotational entropy of the free ligand (-*T*Δ*S*_rot_ and -*T*Δ*S*_trans_ in Table 1). The differences in translational and rotational entropies of the CA-L and CA-OH complexes were negligibly small for all ligands (< 0.04 kcal·mol^− 1^).

**Table 1:** Breakdown of thermal correction contributions in kcal·mol^-1^ for two representative ligands **1** and **23**

Ligand	1	23
Δ*G*_corr_	4.0	7.1
Δ*H*_corr_	-0.4	-0.2
-*T*Δ*S*	4.4	7.3
-*T*Δ*S*_vib_	-1.7	-3.9
-*T*Δ*S*_rot_	4.5	8.0
-*T*Δ*S*_trans_	1.6	3.2

Although the inclusion of thermal effects slightly corrected for the overestimation of binding affinity (6.9 kcal·mol^− 1^ on average), it resulted in a slight decrease in the correlation to experimental binding affinities with *R*^2^ of 0.60 and 0.64, respectively. Clearly, this may be due to several reasons, including the assumptions underlying the RR-QHO approximation (ideal gas, rigid rotor and quasi-harmonic oscillator), the neglect of solvent effects, and the use of a “single-structure” approach which neglects conformational effects. In the next section, we consider the inclusion of solvent effects.

### Consideration of solvent effects

The three-dimensional reference interaction site model (3D-RISM) was employed to account for these solvent effects. Unlike continuum models, it captures the distribution of solvents around the solute, enabling a more detailed description of solute-solvent interactions such as hydrogen bonding. A study by Imai et al. reported that water distribution inside egg white lysozyme predicted with 3D-RISM is in good agreement with experimental observations [72]. 

The addition of solvation free energies to Δ*E*_bind_ greatly improved the MAD between QM/MM (using QM/MM model II) and experimental binding affinity from 36.7 to 5.1 kcal·mol^− 1^. The correction is largely driven by the difference in the solvation free energy between the CA-L and CA-OH complexes. Interestingly, the inclusion of solvation free energies did not improve the correlation with a slight decrease in *R*^2^ from 0.64 to 0.60. Further, the addition of thermal correction to recover the complete solution-phase free energy of binding in Eq. 1 significantly lowered the correlation (*R*^2^ = 0.47) and increased the MAD to 11.6 kcal·mol^− 1^ from 5.1 kcal·mol^− 1^.

These results indicate that inclusion of solvent and thermal effects can improve the agreement between experimental and computed binding energies. However, where trends are concerned, which is the focus of this work, inclusion of these effects has a detrimental effect. As noted above, this may be due to several reasons including the intrinsic errors of the RR-QHO approximation and the 3D-RISM model. For example, an optimized version of 3D-RISM model yielded mean error of 1.5 kcal mol^− 1^ for the calculation of hydration free energies of small neutral solutes; [73] these errors are expected to increase for the large multiply charged enzymes. Nevertheless, significant cancellation of errors is expected using Scheme 1 to compute binding energies.

Another source of error is the use of a “single-structure” approach based on the crystal structure pose which neglects conformational sampling. One remedy could be to perform Boltzmann weighted averaging of QM/MM optimized snapshots obtained from classical MD simulations. As explained in the Introduction, we decided to base the QM/MM calculations on the crystal structure pose because our prior work on QM-cluster models revealed that the rotamers of the amino acid residues in the binding pocket is highly conserved across a wide range of ligands [22]. Furthermore, this work revealed that the increased degrees of freedom, including relaxation of a larger number of MM atoms or increasing the QM region size could contribute significant noise to the binding energy calculation and weaken the correlation with experimental data. These findings do not bode well for doing QM/MM calculations on ensemble of geometries sampled from classical simulations and direct QM/MM dynamics or classical MD FEP simulations may be preferred.

### Using binding poses from molecular docking

The investigation into the various QM/MM parameters have been performed on inhibitors with available crystal structures and experimental binding affinities. A practical application of the QM/MM model would be to screen yet-to-be synthesized inhibitors to help inform and prioritize experimental work. This means that the starting binding pose needs to be obtained from other means such as molecular docking.

Currently, the ligand charges are fitted on geometries optimized with the crystal binding pose as the starting structure. In the absence of crystal structures, alternative ligand conformations will be used for the ligand charge fitting. To assess the sensitivity of partial charges to ligand geometry, charges derived from gas-phase global minima were compared to those optimized from crystal binding poses (B3LYP/6–31+G(d, p)//r^2^SCAN-3c). Charge variations were generally minor, where absolute differences exceeding 0.1 *e* were largely restricted to shifts in urea conformation or intramolecular hydrogen bonding. Consequently, the impact on binding energies (computed on QM/MM model II) was insignificant, with a mean absolute deviation (MAD) of 0.8 kcal·mol^− 1^, although some large flexible ligands showed deviations up to 2.4 kcal·mol^− 1^. Notably, both sets of charges yielded identical correlations (*R*^2^ = 0.64), confirming that global minimum ligand geometries are a valid alternative for ligand charge fitting when crystal structures are unavailable.

**Fig. 7:**
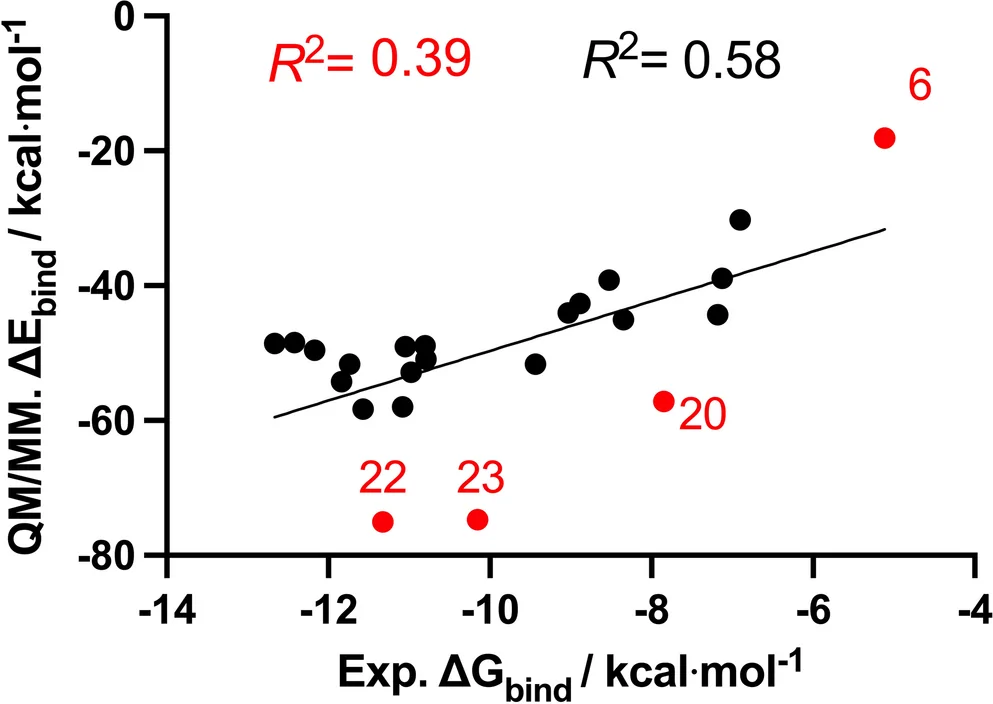
Correlation to experimental binding affinity of QM/MM reoptimized docked poses with (red) and without (black) outliers

Three lowest energy binding poses were re-optimized with QM/MM model II (see Fig. 4) and re-ranked using to their QM/MM energies to locate the lowest energy pose for the binding energy calculation. Figure 7 presents the predicted binding energies using QM/MM optimized docked poses. These results can be directly compared with Fig. 5(b) which are obtained using crystal structure poses. Four ligands (**6**, **20**, **22**, and **23**) appear as clear outliers in the binding affinity results with deviations larger than two times the mean absolute deviation from the fitted trend line.

Ligand **6** was earlier identified as an outlier due to it being chemically distinct (a hydroxamate) from the other ligands in the test set. This difference in functionality (and binding mode) also led to a complication in the docking simulations. The AutoDock4_Zn_ force field is designed to enforce the tetrahedral coordination of the zinc ion by driving an overlap between a dummy atom in the tetrahedral position and the zinc-binding atom of the ligand (typically a deprotonated nitrogen) [27]. This is achieved by applying a strong van der Waals (vDW) potential with a short equilibrium distance between the dummy atom and the zinc-binding atom. However, this design inherently fails for ligand **6**, which is the only hydroxamate (bidentate) ligand. This incompatibility resulted in vastly incorrect binding poses where the benzene ring is incorrectly positioned near the zinc ion.

We attempted to adjust the vdW equilibrium distance between zinc and oxygen of the deprotonated hydroxamate to 2.1 Å (based on average Zn-O distances in the PDB) which recovered the crystallographic bidentate binding mode in one of the docked poses. However, the ligand N-H hydrogen was incorrectly pointing away from the Thr200 where subsequent QM/MM geometry optimization produced the incorrect mondentate binding pose. This example highlights the sensitivity of the QM/MM model to input geometry.

**Fig. 8:**
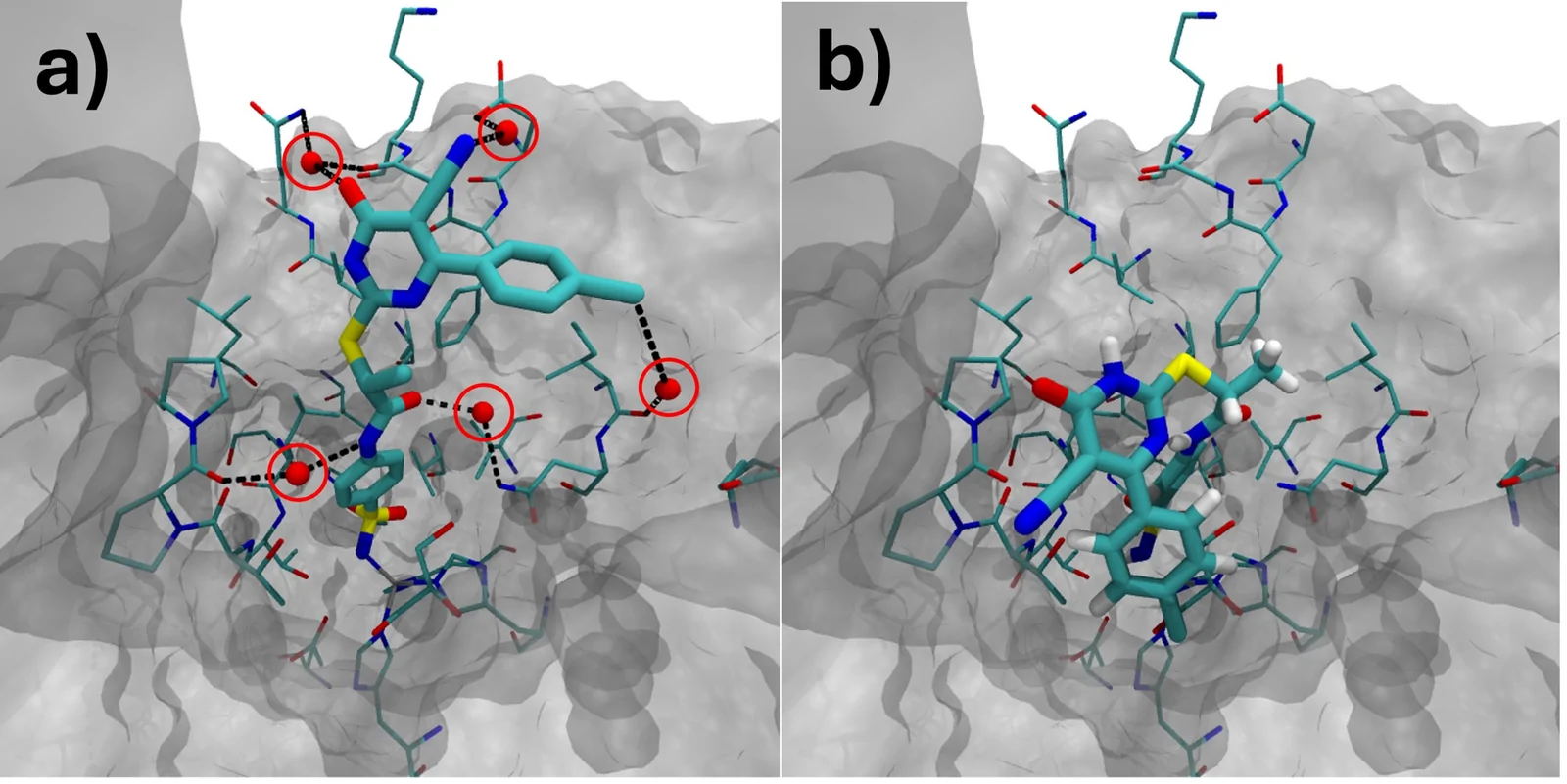
**a **Crystal binding poses with five bridging water molecules represented highlighted with red circles. **b **Lowest energy QM/MM reoptimized docked pose in the gas-phase

The three remaining outliers arose because the model incorrectly predicted the energy of a docked pose (furthest from the crystal pose) as the lowest energy pose. Ligand **22** was analyzed as a case study to investigate the 22.9 kcal·mol⁻¹ energy gap favoring the lowest energy docked pose over the pose that is closest to the crystal structure. The discrepancy was traced to crystallographic waters (Fig. 8) that stabilize the crystal pose via hydrogen bonding though these waters were not included in the molecular docking or QM/MM models.

When these five crystal waters were added to the MM region of both poses (employing a stepwise optimization of hydrogens, then water molecules, then the full relaxed region (a) to preserve crystal coordinates), the energy gap favoring the docked pose decreased to 15.1 kcal·mol⁻¹. Including these waters in the QM region further reduced the gap to 7.8 kcal·mol⁻¹. Finally, applying 3D-RISM solvation to the geometries optimized with QM water reversed the relative stability, making the crystal pose 18.4 kcal·mol⁻¹ more stable than the docked pose. In comparison, application of 3D-RISM on gas-phase structures without explicit water reduced the gap to 5.5 kcal·mol⁻¹ but failed to identify the crystal pose as the energetically more favorable one.

These results suggest that combining explicit active-site waters with 3D-RISM is crucial for accurate prediction of binding pose for large ligands such as **20**–**23** which have tails containing hydrophilic groups that extend to the solvent exposed part of the enzyme. Further work is needed to assess suitable approaches for predicting the placement of these water molecules (e.g. molecular docking with ligand attached water [74] or Rosetta-based PlaceWaters [75] protocol), and how to deal with the varying number of water molecules that will differ for different ligands. This inevitably complicates the calculation of the binding energy using Scheme 1. An alternative is to employ QM/MM molecular dynamics free energy simulations in conjunction with, for example, umbrella sampling to estimate absolute binding energies. However, this approach is significantly more expensive, which prohibits the use of accurate QM levels of theory as well as the examination of larger QM region sizes.

## Conclusion

In this study, we sought to answer the question on whether QM/MM model predictions are systematically improvable in the context of predicting trends in carbonic anhydrase-ligand binding affinities. Interestingly, it appears that increasing the size of the QM region and/or number of MM relaxed atoms nor the inclusion of thermal and solvent effects offered any improvement. At best, we were able to obtain an *R*^2^ that is close to 0.7 for a diverse set of sulfonamide ligands. Increasing QM region size or relaxing more MM atoms appears to introduce more noise due to the increased QM degrees of freedom which slightly weakened the correlation with experimental binding free energies. Incorporation of solvent effects brought the calculated binding energies into closer agreement with experiment; however, due to the relatively small range in the binding free energies (ca. 8 kcal mol^-1^), small errors in the computed binding energies can very easily skew the correlation with experiment.

As noted in the Introduction, ligands **19**–**23** were included to test the limits of the computational models. These larger and conformational flexible molecules posed a challenge for both QM-cluster and QM/MM models presumably due to the role of water molecules that bridge the ligand interactions with the enzyme which also affect their orientational states that are difficult to treat using a single structure static QM/MM approach. Nevertheless, removal of these species resulted in a much higher *R*^2^ of 0.82 for the smallest QM/MM model (Fig. 5a) without the need to consider solvent or thermal effects. This result is significantly better than using docking scores from Autodock Vina or even very recently reported QM/MM metadynamics and classical MD/free energy perturbation simulations [76] which reported an *R*^2^ of 0.7 with experimental binding free energies for a much smaller subset of five structurally simple ligands. Thus, our work here suggests an economical QM/MM approach that may be used to reliably screening relatively small and conformationally rigid ligands.

We further examined an important practical issue concerning the use of binding poses from molecular docking programs such as Autodock Vina where crystal structure data is unavailable. Our study indicates that gas phase QM/MM optimization to re-rank docked poses for use in binding energy calculations is a sound strategy for most of the ligands in our dataset. However, this approach fails abysmally for certain ligands (e.g. ureidosulfonamides) where their interactions with the enzyme involve bridging water molecules. In such cases, we found that the inclusion of MM explicit waters in the binding pocket and bulk solvation effects recovered using the 3D-RISM model is needed to predict the correct crystal binding pose.

In future work, it would be interesting to examine approaches for predicting the placement of active site waters as well as classical and QM/MM MD/FEP approaches based on the same dataset that directly incorporate conformational, solvent and thermal effects. Future work should also examine the system-dependence of our findings by studying other types of proteins and reaction types. Nevertheless, due to the high degree of structural homology between various isoforms of carbonic anhydrase, which are distinct drug targets for different diseases, we envisage that the findings here will be directly transferable to these other isoforms and possibly related metalloenzymes such as MMPs and HDAC. Our work presents a useful baseline comparison for evaluating the cost and accuracy of more sophisticated simulation protocols in the context of carbonic anhydrase as a prominent drug target.

## Supplementary Information

Supplementary Material 1: Additional Figures and Tables to support discussions.

Supplementary Material 2: Excel spreadsheet containing the raw QM and QM/MM energies.

Supplementary Material 3: Example QM/MM input and output files.

## Data Availability

The detailed steps in utilizing the TAO package for QM/MM input file setup and the TAO package are freely available at https://s2.smu.edu/ptao/software/taopackage/TAOpackage.html. The H++ server used to protonate the apoenzyme can be found at http://newbiophysics.cs.vt.edu/H++/. The AmberTools22 package is freely available at https://ambermd.org/AmberTools.php. All binding affinity data presented in this work are available in the Supporting_Information.xlsx file. The QM/MM procedure and example input files including the in-house external Gaussian python script are provided in the zip folder.
