# Supplementary material for: Are QM/MM models systematically improvable? A case study on carbonic anhydrase binding affinities
Source: J Comput Aided Mol Des. 2026 Oct 7;40(1):249. doi: 10.1007/s10822-026-00954-8 (PMC13642402; doi:10.1007/s10822-026-00954-8)
Supplement: Supplementary file 1 [file 10822_2026_954_MOESM1_ESM.pdf]

Are QM/MM Models Systematically Improvable? A Case Study on Carbonic Anhydrase Binding Affinities

Haedam Mun, Junming Ho*

School of Chemistry, The University of New South Wales, Sydney, NSW 2052, Australia

*Corresponding author email: junming.ho@unsw.edu.au

Supporting Information

RESP charges generated at different levels of theory for ligand 2 and Zn(His)$_3$ core

Table S1. The RESP charges of ligand 2 generated at different levels of theory.

Ligand 2	HF			B3LYP		
	6-31G(d)	6-31+G(d)	6-31+G(d, p)	6-31G(d)	6-31+G(d)	6-31+G(d, p)
H1	0.11	0.11	0.10	0.08	0.08	0.08
H2	0.11	0.12	0.11	0.09	0.09	0.09
H3	0.13	0.13	0.13	0.10	0.11	0.11
H4	0.13	0.13	0.13	0.10	0.11	0.11
H5	0.11	0.12	0.11	0.09	0.09	0.09
H6	0.30	0.30	0.30	0.27	0.28	0.28
C1	-0.13	-0.12	-0.12	-0.12	-0.10	-0.10
C2	-0.18	-0.20	-0.20	-0.16	-0.18	-0.17
C3	-0.08	-0.08	-0.08	-0.07	-0.08	-0.08
C4	-0.01	0.00	0.00	0.02	0.03	0.02
C5	-0.08	-0.08	-0.08	-0.07	-0.08	-0.08
C6	-0.18	-0.20	-0.20	-0.16	-0.18	-0.17
N1	-0.92	-0.97	-0.97	-0.79	-0.88	-0.88
O1	-0.64	-0.69	-0.68	-0.54	-0.62	-0.62
O2	-0.64	-0.69	-0.68	-0.54	-0.62	-0.62
S1	0.99	1.12	1.11	0.71	0.95	0.95

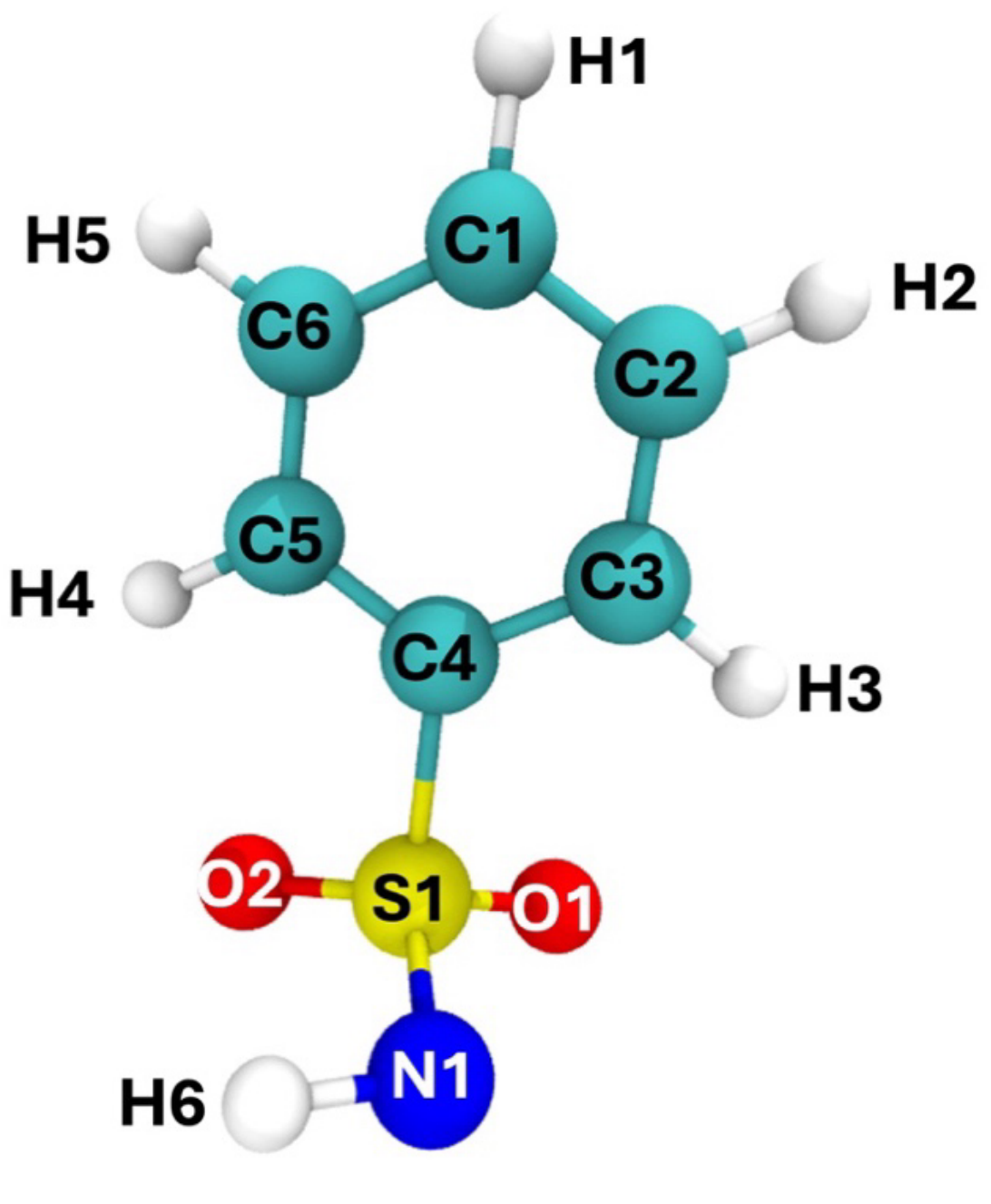

Figure S1. Atom indices of ligand 2

Table S2. The RESP charges of $Zn(His)_3$ core generated at different levels of theory. The zinc and coordinating nitrogens are highlighted.

	HF			B3LYP		
	6-31G(d)	6-31+G(d)	6-31+G(d,p)	6-31G(d)	6-31+G(d)	6-31+G(d,p)
C	0.17	0.17	0.17	0.17	0.17	0.18
H	0.03	0.03	0.03	0.03	0.03	0.03
H	0.03	0.03	0.03	0.03	0.03	0.03
C	-0.05	-0.05	-0.06	-0.05	-0.05	-0.06
N	-0.07	-0.06	-0.05	-0.03	-0.04	-0.03
H	0.31	0.31	0.31	0.30	0.30	0.30
C	0.05	0.04	0.04	0.03	0.02	0.02
H	0.21	0.21	0.21	0.19	0.19	0.18
N	-0.51	-0.46	-0.46	-0.43	-0.39	-0.39
C	-0.06	-0.08	-0.08	-0.03	-0.05	-0.05
H	0.18	0.18	0.18	0.15	0.16	0.16
C	0.23	0.24	0.25	0.24	0.25	0.25
H	0.00	0.00	0.00	0.01	0.00	0.00
H	0.00	-0.01	-0.01	0.00	-0.01	-0.01
C	0.00	0.00	0.00	0.00	0.00	-0.01
N	-0.17	-0.18	-0.17	-0.13	-0.15	-0.14
H	0.36	0.36	0.36	0.34	0.35	0.35
C	0.04	0.04	0.04	0.02	0.02	0.02
H	0.22	0.22	0.22	0.20	0.20	0.19
N	-0.41	-0.38	-0.38	-0.35	-0.32	-0.32
C	-0.18	-0.19	-0.19	-0.14	-0.15	-0.14
H	0.17	0.16	0.16	0.14	0.14	0.14
C	0.29	0.30	0.30	0.28	0.29	0.29
H	-0.12	-0.13	-0.13	-0.12	-0.12	-0.12
H	-0.02	-0.02	-0.02	-0.02	-0.02	-0.02
C	0.08	0.07	0.06	0.08	0.07	0.07
N	-0.47	-0.42	-0.42	-0.41	-0.36	-0.36
C	0.04	0.03	0.03	0.03	0.01	0.01
H	0.20	0.20	0.20	0.18	0.18	0.18
N	-0.08	-0.07	-0.07	-0.06	-0.06	-0.05
H	0.34	0.34	0.34	0.33	0.33	0.32
C	-0.24	-0.25	-0.24	-0.18	-0.19	-0.19
H	0.25	0.25	0.25	0.22	0.22	0.22
Zn	1.16	1.11	1.10	1.01	0.95	0.95

Table S3. ΔE(MM) associated with the three QM/MM model **II** with relaxed MM region according to criteria (a), (b), and (c). See Equations (2) to (4) in main text.

	ΔE(MM) kcal mol^{-1}		
Ligand	(a)	(b)	(c)
1	-4.4	-3.2	-2.2
2	-13.3	-12.0	-11.3
3	-15.6	-14.3	-12.5
4	-15.6	-14.1	-13.2
5	-14.7	-12.8	-10.9
6	0.2	1.0	3.1
7	-16.9	-17.3	-16.4
8	-18.3	-18.7	-17.9
9	-19.3	-19.4	-18.7
10	-20.4	-20.9	-19.9
11	-24.3	-24.6	-22.6
12	-28.0	-27.1	-24.0
13	-20.7	-19.6	-17.3
14	-24.6	-23.2	-21.2
15	-14.8	-15.2	-14.8
16	-19.4	-19.6	-18.6
17	-12.8	-11.1	-10.2
18	-6.7	-8.3	-8.0
19	-26.5	-25.5	-21.3
20	-21.9	-21.8	-21.1
21	-32.1	-28.6	-26.8
22	-38.3	-37.6	-33.4
23	-46.7	-45.8	-46.3
standard deviation	10.4	10.2	10.0

Table S4. QM/MM binding energies (ΔE_{gas}) in kcal mol^{-1} computed with $Zn(His)_3$ and ligand charges generated at different levels of theory.

	HF			B3LYP		
Ligand	6-31G(d)	6-31+G(d)	6-31+G(d,p)	6-31G(d)	6-31+G(d)	6-31+G(d,p)
1	-46.9	-47.7	-47.6	-45.8	-46.8	-46.7
2	-45.8	-46.6	-46.5	-44.8	-45.8	-45.8
3	-47.3	-48.1	-48.1	-46.3	-47.4	-47.3
4	-53.6	-54.9	-54.9	-51.2	-53.1	-53.0
5	-50.1	-50.8	-50.8	-48.9	-50.0	-49.9
6	-41.4	-42.2	-42.1	-40.5	-41.6	-41.5
7	-47.1	-48.1	-48.1	-45.8	-47.2	-47.2
8	-27.0	-27.8	-27.7	-25.8	-27.0	-26.9
9	-46.8	-47.7	-47.6	-45.7	-46.8	-46.7
10	-23.4	-23.8	-23.7	-24.2	-24.5	-24.4
11	-40.7	-41.7	-41.6	-39.4	-40.8	-40.7
12	-47.3	-48.2	-48.2	-46.1	-47.4	-47.3
13	-40.4	-41.1	-41.1	-39.5	-40.4	-40.4
14	-51.8	-52.9	-52.1	-49.8	-51.5	-51.4
15	-57.1	-57.8	-57.8	-55.3	-56.5	-56.4
16	-36.9	-37.4	-37.4	-36.7	-37.4	-37.4
17	-37.9	-38.6	-38.5	-36.7	-37.7	-37.6
18	-50.0	-51.0	-50.9	-48.2	-49.6	-49.5
19	-43.6	-44.2	-44.1	-42.5	-43.3	-43.2
20	-41.4	-42.2	-42.1	-40.6	-41.6	-41.5
21	-49.9	-51.0	-51.0	-48.4	-49.9	-49.9
22	-56.7	-57.5	-57.4	-54.0	-55.6	-55.5
23	-51.2	-52.0	-51.9	-49.3	-50.6	-50.5
R^2	0.64	0.64	0.64	0.64	0.65	0.65

Electrostatic embedding vs mechanical embedding for energy calculations.

Comparison of ME and EE calculations utilized the ONIOM(B3LYP-D3(BJ)/def2-TZVPP:AMBER*) energies computed using Gaussian16. This is due to complications with exporting MM point charges into the ORCA external program for the EE calculations. The comparison between ME and EE energies were made for all three QM/MM models to investigate dependence on the QM region size (position of the QM/MM boundary). The mean absolute deviation (MAD) in binding affinities between ME and EE differed on average 4.0, 1.9, and 6.6 kcal·mol^{-1} for QM/MM model **I**, **II**, and **III**, respectively. The correlation coefficients showed a smaller difference with EE consistently resulting in a slight decrease in the correlation.

Table S5. Pearson correlation coefficient (R^2) between QM/MM-ME, QM/MM-EE (ΔE_{gas}) binding free energies with experimental binding free energies. Energies computed at the ONIOM(B3LYP-D3(BJ)/def2-TZVPP:AMBER*) level on ONIOM(r^{2}SCAN-3c:AMBER*) geometries for the three QM/MM models.

QM/MM model	Mechanical Embedding	Electrostatic Embedding
**I**	0.70	0.64
**II**	0.66	0.61
**III**	0.63	0.61

References

(1) Ho, J.; Yu, H. B.; Shao, Y. H.; Taylor, M.; Chen, J. B. How Accurate Are QM/MM Models? *J. Phys. Chem. A.* **2024**, *129* (6), 1517–1528. DOI: 10.1021/acs.jpca.4c06521.
(2) Hu, L. H.; Söderhjelm, P.; Ryde, U. On the Convergence of QM/MM Energies. *J. Chem. Theory Comput.* **2011**, *7* (3), 761–777. DOI: 10.1021/ct100530r.
(3) Sumowski, C. V.; Ochsenfeld, C. A Convergence Study of QM/MM Isomerization Energies with the Selected Size of the QM Region for Peptidic Systems. *J. Phys. Chem. A.* **2009**, *113* (43), 11734–11741. DOI: 10.1021/jp902876n.
(4) Wang, B.; Truhlar, D. G. Combined Quantum Mechanical and Molecular Mechanical Methods for Calculating Potential Energy Surfaces: Tuned and Balanced Redistributed-Charge Algorithm. *J. Chem. Theory Comput.* **2010**, *6* (2), 359–369. DOI: 10.1021/ct900366m.
(5) Lin, H.; Truhlar, D. G. Redistributed Charge and Dipole Schemes for Combined Quantum Mechanical and Molecular Mechanical Calculations. *J. Phys. Chem. A.* **2005**, *109* (17), 3991–4004. DOI: 10.1021/jp0446332.
